# Measuring Duchenne muscular dystrophy impact: development of a proxy-reported measure derived from PROMIS item banks

**DOI:** 10.1186/s13023-021-02114-7

**Published:** 2021-11-22

**Authors:** Carolyn E. Schwartz, Roland B. Stark, David Cella, Katrina Borowiec, Katherine L. Gooch, Ivana F. Audhya

**Affiliations:** 1 DeltaQuest Foundation, Inc., 31 Mitchell Road, Concord, MA 01742 USA; 2 Departments of Medicine and Orthopaedic Surgery, Tufts University Medical School, Boston, MA USA; 3 Department of Medical Social Sciences, Feinberg School of Medicine at Northwestern University, Chicago, IL USA; 4 Department of Measurement, Evaluation, Statistics, and Assessment, Boston College Lynch School of Education and Human Development, Chestnut Hill, MA USA; 5 Sarepta Therapeutics, Cambridge, MA USA

**Keywords:** Duchenne muscular dystrophy, Disability, Neuromuscular, Proxy measurement, Validation, Item response theory, Classical test theory

## Abstract

**Background:**

Person-reported outcomes measurement development for rare diseases has lagged behind that of more common diseases. In studies of caregivers of patients with rare diseases, one relies on proxy report to characterize this disability. It is important to measure the child’s disability accurately and comprehensively because it affects caregiver burden. We aimed to create a condition-specific caregiver proxy-report measure for Duchenne Muscular Dystrophy (DMD) in order to understand the impact of DMD on the caregiver. Drawing on relevant item banks from the Patient-Reported Outcome Measurement Information System (PROMIS), we sought to confirm their reliability and validity in the target sample of DMD caregivers.

**Methods:**

This web-based study recruited DMD caregivers via Rare Patient Voice, patient-advocacy groups, and word of mouth. Recruitment was stratified by age of the caregiver’s child with DMD, which broadly represents stages of DMD progression: 2–7, 8–12, 13–17, and > 18. Telephone interviews with DMD parent-caregivers pretested possible measures for content validity. The web-based study utilized an algorithm to categorize respondents’ ambulatory status for tailored administration of PROMIS Parent-Proxy items as well as some new items developed based on caregiver interviews. Item response theory analyses were implemented.

**Results:**

The study sample included 521 DMD caregivers representing equally the four age strata. The proxy-report measure included the following domains: fatigue impact, strength impact, cognitive function, upper extremity function, positive affect, negative affect, sleep-device symptoms, and mobility. The first five domains had strong psychometric characteristics (unidimensionality; acceptable model fit; strong standardized factor loadings; high marginal reliability). Negative Affect, covering anger, anxiety, depressive symptoms, and psychological stress, fit a bifactor model with good model fit, high marginal reliability, and strong factor loadings. The Sleep-device symptoms domain was not unidimensional, and the mobility domain did not have a simple structure due to residual correlations among items at opposite end of the mobility-disability continuum. These two domain scores were retained as clinimetric indices (i.e., uncalibrated scales), to achieve the overall goal of having a content-valid DMD-specific measure across all stages of disease severity.

**Conclusions:**

The present study derived a DMD-specific proxy-report measure from PROMIS item banks and supplemental items that could potentially be utilized in caregiver research across all stages of the care recipient’s DMD. Future research will focus on assessing the responsiveness and validity of the measure over time and its comparison to DMD patient self-report.

## Introduction

The introduction of person-reported outcomes (PROs) over the past few decades has facilitated clinical research in many ways. PROs make it increasingly feasible for the patient’s voice to be heard in important studies of great relevance to them [1–3]. Similarly, when using parent-proxy report, PROs enable the parent to reflect their child’s experience when the child is too young or not able to complete a survey him/herself. Further, in order to understand the impact of caregiving, it would be critical to consider the multiple domains of their care recipient’s disability. Some domains may be easier to address and manage, whereas others may present more stressful and distressing challenges.

For conditions with high prevalence, condition-specific PROs and proxy measures were often developed early in the field of quality-of-life (QOL) research. For example, cancer measures were among the first to be developed, with site-specific modules created in the US [4] and Europe [5] complementing general measures. For less prevalent conditions, the early approach was to use a more generic measure, such as the SF-36™ [6, 7] for primary care [8], end-stage renal disease [9], arthritis [10], etc.; or, in some cases, to add some condition-specific items to fit the purpose (e.g., multiple sclerosis [11], epilepsy [12]). These early measures were later complemented by fully disease-specific PRO and proxy-report measures that tapped the relevant domains for the target condition (e.g., multiple sclerosis [13], arthritis [14, 15], epilepsy [16]).

For rare diseases such as Duchenne Muscular Dystrophy (DMD), however, PRO and proxy measurement development has typically been slower. Perhaps due to the substantial effort to undertake recruitment of patients with rare conditions, this measurement gap remains a concern. Research studies that fail to utilize content-valid PRO or proxy measures risks missing important effects of interventions, medications, or developmental changes. Other DMD-specific measures have been proposed, such as the PedsQL DMD module [17], the MDCHILD out of Canada [18], and the DMD-QoL work as part of Project HERCULES in the UK [19]. A recent review of QOL measures in DMD revealed that evidence was lacking on the content and structural validity of the PedsQL DMD module, and the MDCHILD, which had been used on but not validated for people with DMD, and that no measures for adults with DMD had a sufficient evidence base to support recommendation [20]. The DMD-QOL was developed after the above review, and lists the above findings as part of its motivation for developing a new PRO.

The present work thus sought to derive a condition-specific proxy-reported measure of DMD disability. This measure was intended to support an in-depth investigation of the impact of DMD on caregiver QOL, work productivity, ambitions, and financial well-being. By measuring via proxy report the DMD patient’s level and scope of disability, we hoped to gain a better understanding of the context of caregiving. Accordingly, patient disability (the target construct) was deemed relevant to caregiver impact,^1^ which was the focus of the larger investigation from which the present study grew. The “DMD disability” construct is defined to be the patient’s level of functioning in domains affected by DMD, such as upper and lower extremity function, strength, mobility, fatigue, cognitive function, and affect. Such a construct is clearly within the purview of QOL measures, and would constitute a disease-specific measure for DMD.

We thus sought to derive a reliable and valid proxy measure by drawing on the available and sophisticated National Institutes of Health-funded resource for clinical researchers: the Patient-Reported Outcome Measurement Information System (PROMIS) [22–24]. The advantages of building from a well-characterized item bank are numerous [25], including utilizing well-honed items that tap a well-defined construct, have known item characteristics in other study populations, and can facilitate comparisons across study populations. The fit of the PROMIS item banks to the DMD context is, however, unknown. Accordingly, the present work sought to address that gap.

DMD is a progressive rare neuromuscular disorder that occurs primarily in males in 16–20 per 100,000 live births in the United States and United Kingdom [26–28]. DMD is usually diagnosed by age 5. The disorder presents as delayed development that includes motor difficulties [29] and may include cognitive impairment and attention deficit disorders [30]. Progressive muscle weakness leads to loss of ambulation, upper-limb function problems, and comorbid conditions such as scoliosis and muscular contractures [29]. As DMD continues to progress, patients experience life-threatening heart and lung conditions [31], and face profound uncertainty regarding lifespan, typically dying in their late 20 s to early 30 s [31]. While disability progression is heterogenous across individuals, on average the trajectory can be categorized in age-related stages: ambulatory (up to age 7), transitional (ages 8–12), and non-ambulatory (≥ age 13). Because disability worsens as patients age into adulthood [32], we sought to create a proxy-report measure so that DMD caregivers could provide consistent information about their child’s functioning, regardless of the child’s age, level of DMD progression, or cognitive function. This approach was chosen to avoid issues such as method variance [33] in the source of disability assessment across care-recipients’ ages and across raters.

## Methods

### Sample and procedure

This study recruited participants via Rare Patient Voice, patient-advocacy groups, and word of mouth. Eligible participants were age 18 or older, able to complete an online questionnaire, and were providing caregiving support to a family-member with DMD at least two years old, usually their son. This survey was administered through the HIPAA-compliant, secure Alchemer engine (www.alchemer.com). Recruitment was stratified by age of the caregiver’s child with DMD: 2–7, 8–12, 13–17, and >  = 18. These strata broadly correspond to the disease-related phases of progression [31]: ambulatory phase (age 2–7), transitional phase (up to age 12), and non-ambulatory phase (age ≥ 13), with increasing dependence and involvement of other systems as the person ages into adulthood (age ≥ 18). If caregivers had more than one person with DMD for whom they were providing caregiving support, they were asked to report on the eldest or most disabled person with DMD (the index patient). Caregivers were paid $75 honoraria to compensate them for their time completing the survey. The protocol was reviewed and approved by the New England Independent Review Board (NEIRB #20201623), and all participants provided informed consent prior to beginning the survey.

### Conceptual development

Telephone interviews were conducted with DMD caregivers to pretest possible measures of DMD disability (i.e., symptoms and impact). In this context, we sought to further scale development, rather than to engage in extensive qualitative research on the participants or on the construct of disability. We relied on the extensive foundational work done by the PROMIS Health Measures collaborative group (www.promishealth.org), and focused on pretesting items in the selected domains thought most applicable to DMD by several of the authors (CES, KG, and IA).

Candidate interviewees were first identified through Rare Patient Voice, which provided the ages of the caregivers’ DMD children. Interviewees were then recruited using a stratified random sampling to represent evenly the three age strata of interest for our research. Two rounds of interviews (n = 9 and 6, respectively) were conducted and documented by the first author. Feedback from the first round informed the materials assessed in the second round. While we aimed to balance males and females in the interview group, the numbers responding were 3 and 12, respectively.

Prior to the interview, participants completed a brief online survey that included the questions from PROMIS item banks and/or short forms being considered for inclusion on the basis of a literature review on DMD disability. The interview then discussed the survey items, asking whether they captured relevant aspects of the care recipient’s disability, whether the items were understandable, and whether additional domains or content should be included. Two important additions that followed from these interviews was the creation of the sleep disturbance items, and inclusion of both positive and negative affect domains. Numerous other changes were made to individual items in various domains, such as revising response options for applicability and for consistency across domains, changing “kids” to “people”, including peer-relationship domains, asking about the full range of mobility impairment so that more of the experience of DMD was reflected. With all of these changes, we aimed to reflect accurately the disability experiences of DMD, and implicitly to acknowledge the challenges faced by these families.

Based on content identified in these interviews, we selected 11 PROMIS [23, 24] parent-proxy short forms and/or item banks: mobility, sleep disturbance, fatigue, strength impact, upper extremity, cognitive function, positive affect, anger, anxiety, depressive symptoms, and psychological stress. A small set of items from each of these latter four domains were selected to tap ‘negative affect.’

### Item development

#### PROMIS items

Using item calibrations provided by the PROMIS Assessment Center, we further selected a set of items within each domain deemed relevant to DMD. With permission from the Center, we made adjustments to items to reference the name of the person with DMD rather than “your child,” to accommodate the fact that the caregiver might be reporting on someone ranging in age from early childhood to middle-age and that may not be their child. In addition, again with permission, we changed response options for the strength-impact domain to be the same as for the upper-extremity domain for the sake of consistency within the measure, and we allowed respondents to select “do not know/prefer not to answer” on all items, to avoid survey attrition.

#### New items

We wrote new items to tap concepts not adequately captured in the PROMIS parent-proxy items, specifically peer-group relations (adapted self-report PROMIS items to proxy-report), use of a medical scooter for mobility, and aspects of sleep-disturbance related to medical devices (e.g., leg braces, continuous positive airway pressure machine). Again, all new items were framed from the perspective of the parent-proxy.

#### Tailored administration

The resulting measure of eight domains included 54 items, but some would only be relevant to a subset of the caregivers. For example, items related to low levels of ambulation disability would only be relevant to caregivers of ambulatory DMD patients, whereas items related to later stages of ambulation disability would only be relevant to caregivers of non-ambulatory DMD patients. Presenting such irrelevant items might also be upsetting to the respondent. We thus categorized items as to whether they should be seen across all levels of DMD—i.e., Ambulatory (A), Transitional (T), Non-ambulatory (N) or only by a subset (i.e., A, AT, ATN, TN, or N). We then utilized a method developed earlier by members of our group for creating disability-specific short forms for multiple sclerosis [34, 35]. Accordingly, we utilized the Lowes Lab Ambulatory Status Algorithm [36] (“Lowes Algorithm”), a recognized branching logic to identify the questions appropriate to the child’s level of disability, and to effectively reduce measure length. This approach was more feasible for our assessment context than the PROMIS computerized adaptive test because the survey engine was not compatible with the PROMIS Application Programming Interface, which such a test would have required.

For ease of interpretation, ambiguous domain names were modified such that ‘impact’ indicated a ‘higher-is-worse’ score, whereas ‘function’ indicated a ‘higher-is-better’ score (e.g., “strength” became “strength impact” and “cognitive” became “cognitive function”).

### Other measures

The *Lowes Lab Ambulatory Status Algorithm *[36] was used to categorize caregiver respondents for a tailored item administration for the mobility and sleep-disturbance domains. Respondents were asked three to five questions in order to categorize their child with DMD as either ambulatory, transitional, or non-ambulatory. These questions were set up as branching logic in the survey engine.

*Demographic characteristics* included year of birth, gender, cohabitation/marital status, employment status, ethnicity, race, education, height, weight, difficulty paying bills, with whom the person lives, smoking status, and whether they received help to complete survey.

### Statistical analysis

For items with missing data due to the skip logic of the tailored administration, missing data were imputed to reflect the individual’s level of functioning known from the Lowes Algorithm. For example, if the individual was categorized as Non-Ambulatory, then an item related to being able to walk a mile was recoded from missing to ‘not able to do’. Such imputation was applicable only for certain mobility and sleep items.

Psychometric analysis included item response theory (IRT) modeling [37, 38]. Because we started with a previously-validated set of PROMIS items, the construct validity has already been demonstrated. Further, the content validity of the selected items/domains was established on the basis of the pretesting interviews with caregivers. The focus of analysis within a domain was on selecting the best set of unidimensional items so that the final scale would be comprehensive, not contain redundant items, and have high internal consistency reliability.

Confirmatory factor analysis (CFA) using Mplus version 8.4 [39] was implemented in all eight domains: fatigue impact, strength impact, negative affect, sleep-device symptoms, cognitive function, upper extremity function, positive affect, and mobility. CFAs were implemented in an iterative fashion to identify items that should be dropped due to high residual correlations as reflected in the modification indices. We examined model-fit statistics, standardized factor loadings, marginal reliability, and modification indices. All CFA analyses used weighted least squares mean- and variance-adjusted estimation, and used as default listwise deletion [39]. Sensitivity analyses compared results with and without the imputation described above. Model fit focused on the Root Mean Square Error of Approximation (RMSEA), the Comparative Fit Index (CFI), and the Tucker-Lewis Index (TLI). While we would generally use standard criteria for good fit (i.e., RMSEA < 0.08, CFI ≥ 0.90, TLI ≥ 0.95) [40], it has been shown that in CFA the RMSEA criterion depends substantially on sample size, number of items in a scale, and meeting distributional assumptions that are often not met with PRO data [41, 42]. Thus, the RMSEA cut-offs are somewhat arbitrary, and assessing whether an item bank is “unidimensional enough” for modeling using IRT requires a balanced consideration of all three fit statistics [41]. Using IRT PRO version 3.1 [43], we then implemented IRT analyses, fitting a graded-response model [44] using marginal maximum likelihood so that all response patterns were analyzed whether data were missing or otherwise. Each IRT model computed slopes, intercepts, thresholds, item information functions, item trace lines, and the marginal reliability of the scaled scores. It also yielded an IRT scoring table based on the summed score for each domain. IRT PRO and flexMIRT® [45] were used to compute a bifactor graded response model [46, 47] when relevant.

Scoring options were created to increase the accessibility of the DMD Impact Measure derived from PROMIS parent-proxy item banks. End-users could choose between one method based on simple sums, and another based on IRT-based T-scores. An application of the derived measures’ scores was shown using a box-and-whiskers plot for each of the eight domain T scores within each age stratum (2–7, 8–12, 13–17, and 18 +). Univariate Analysis of variance (uni-ANOVA) models computed using SPSS tested for age-related group differences in each of the eight domains.

Descriptive analyses were done using IBM SPSS version 27 [48].

## Results

### Sample

The study sample included 521 DMD caregivers representing equally four age strata: age 2–7, 8–12, 13–17, and 18 or older. The caregiver sample had a mean age of 41.5, and 76% were female (Table 1). The sample was 84% white and 9% black; 11% were Hispanic. The sample was drawn from the contiguous United States, with a larger proportion in the South Atlantic (24%) than other regions. Eighty-six percent of respondents were married or in a domestic partnership. Over half of the sample was employed, about a third of whom worked full-time. The median level of education was a two-year university degree. Caregivers had an average of 1.4 comorbid health conditions out of 15 presented, with the most prevalent being back pain, depression, insomnia, and arthritis. Most were never-smokers, and the average body mass index reflected being overweight.

**Table 1:** Descriptive Statistics of Caregivers (N = 521)

	Mean	SD
Variable		
Age	41.5	8.9
Range	21–72	
Gender		
Male	126	24
Female	395	76
Race		
Black	46	9
White	437	84
Other	38	7
Hispanic ethnicity		
Yes	57	11
No	445	85
Missing	19	4
Marital status		
Never married	30	6
Married	413	79
Cohabitation/domestic partner	37	7
Separated	11	2
Divorced	22	4
Widowed	5	1
Missing	3	1

	Frequency	%
US Region		
South Atlantic	127	24
Pacific	85	16
East North Central	67	13
Middle Atlantic	58	11
West South Central	42	8
East South Central	25	5
Mountain	25	5
West North Central	25	5
New England	18	3
Non-Contiguous	1	0
Missing	48	9
Comorbidities, out of 15 presented (mean, sd)	1.4	1.7
Range	0–9	
Body Mass Index (mean, sd)	27.1	6.1
Range	16.6–40.0	
Specific comorbidities*		
Arthritis	72	14
Asthma	47	9
Back Pain	172	33
Cancer now or in the past	18	4
Depression	127	24
Diabetes	19	4
Heart Disease	11	2
High Blood Pressure	45	9
Insomnia	108	21
Kidney Disease	3	1
Liver Disease	5	1
Lung Disease	3	1
Stroke	3	1
Ulcer or Stomach Disease	17	3
Other	64	12
Smoking status		
Never Smoked	416	80
Used to Smoke	49	9
Some Days Currently	22	4
Every Day Currently	31	6
Missing	3	1
Work status		
Employed	289	55
Unemployed	193	37
Retired	10	2
Disabled due to medical condition	9	2
Missing	20	4
Hours Worked per Week		
Does not apply	231	44
< 20	15	3
20–29	39	7
30–39	72	14
40 +	164	31
Level of education		
Less than 12th grade	6	1
High school diploma	56	11
Technical (Vocational) degree	62	12
Some college	88	17
2-year University degree	79	15
4-year University degree	154	30
Masters degree	47	9
Doctoral degree	5	1
Missing	24	5

*A non-response was counted as the absence of the comorbidity in question

Caregivers reported providing support to one to five people with DMD (mean = 1.1, SD = 0.4), of whom up to three were their children (Table 2). Families had an average of two people other than the caregiver providing this support. Caregivers were almost all (97%) parents of the DMD index person (Table 2). The index person with DMD had a mean age of 12.9 and had an average of 1.6 comorbidities, the most prevalent of which were anxiety, learning disabilities, attention-deficit, scoliosis, sleep disorder, overweight, and depression. According to the Lowes Algorithm, 29% of the sample was ambulatory, 24% transitional, and 42% non-ambulatory (5% were missing information).

**Table 2:** Descriptive statistics of caregiver context and DMD care recipients (N = 521)

	Mean	SD
Variable		
Number of People with DMD Caring for	1.1	0.4
Range	1–5	
Number of Children	1.9	1.0
Range	1–8	
Number of Children with DMD	1.1	0.3
Range	0–3	
Number of Supports Living in the Home	2.1	0.8
Range	0–3	
Age	12.9	6.6
Range	2–42	
Years Cared for by this Caregiver	11.2	7.0
Range	0–42	

	Frequency	%
Caregiver's Relationship to DMD Index Person		
Parent	549	97
Sibling	3	1
Other Relative	9	2
Paid Caregiver	0	0
Other	5	1
Comorbidities, out of 11 presented	1.6	1.8
Range	0–9	

	Frequency	%
DMD treatment		
Deflazacort	216	41
Exondys 51	124	24
Prednisolone	101	19
Other	79	15
Missing	1	0
Lowes lab ambulatory status algorithm category		
Non-ambulatory	217	42
Transitional	127	24
Ambulatory	150	29
Missing	27	5
Specific comorbidities*		
Anxiety	188	36
Asthma	42	8
ADD or ADHD	85	16
Autism spectrum disorder	40	8
Depression	71	14
Diabetes	14	3
Epilepsy	14	3
Overweight	94	18
Learning disabilities	122	23
Scoliosis	81	16
Sleep disorder	82	16

*A non-response was counted as the absence of the comorbidity in question

### Psychometric results

All domains’ descriptive statistics are shown in Table 3. Results of the CFAs showed acceptable unidimensional model fit (Table 4), strong standardized factor loadings (Table 5), and high marginal reliability (Table 4) for the five domains of fatigue impact, strength impact, upper extremity function, cognitive function, and positive affect. Negative Affect fit a bifactor model, which is to be expected since its items were drawn from varied item banks covering anger, anxiety, depressive symptoms, and psychological stress. Negative-affect factor loadings were constrained to equality for one specific factor with only two items. The General factor from this bifactor model had good model fit, high marginal reliability, and strong factor loadings (Tables 4 and 5). Item trace lines within IRT-scored domains suggested that scores reflect the full range of the corresponding latent variable (data not shown). The length of this tailored measure ranges from 48 to 50 items, depending on the level of ambulation disability in the DMD index person (Table 3).

**Table 3:** Descriptive statistics of PROMIS parent proxy domains (N = 521)

Domain	Tailored administration?	No. items	Min	Max	Mean	SD	Skewness	Correlation between Sum Score and IRT T-Score
Fatigue Impact	No	5	34.00	73.00	50.79	9.42	−0.33	0.98
Strength Impact	No	4	35.00	68.00	50.13	9.28	0.03	0.99
Negative Affect	No	9	31.77	75.83	50.32	9.39	−0.16	0.98
Sleep-Device Symptoms*	Yes	4	4.00	18.00	8.77	3.68	0.30	NA
Cognitive Function	No	9	22.00	63.00	50.30	9.41	−0.07	0.95
Upper Extremity Function	No	5	34.00	66.00	50.18	9.60	−0.13	0.98
Positive Affect	No	4	21.00	68.00	50.18	9.37	−0.47	0.99
Mobility*	Yes	13	13.00	65.00	32.47	13.76	0.50	NA
Total number of items across domains for tailored administration								
Ambulatory		50						
Transitional		51						
Non-ambulatory		48						

*Clinimetric index

**Table 4:** Model fit statistics of PROMIS parent proxy domains

Domain	No. items	RMSEA	90% CI, LL	90% CI, UL	CFI	TLI	Marginal reliability
Fatigue impact	5	0.095	0.061	0.133	0.998	0.997	0.92
Strength impact	4	0.070	0.022	0.131	0.999	0.997	0.87
Negative affect^†^	9	0.070	0.053	0.089	0.994	0.989	0.88
Sleep-device symptoms*	4	0.140	0.089	0.198	0.971	0.914	0.75
Cognitive function	9	0.070	0.022	0.131	0.999	0.997	0.89
Upper extremity function	5	0.110	0.078	0.145	0.999	0.997	0.91
Positive affect	4	0.078	0.028	0.135	0.999	0.997	0.87
Mobility*	13	0.145	0.133	0.158	0.987	0.983	0.93

^†^Bifactor model

*Clinimetric index

**Table 5:** DMD disability domains: item content and factor loadings where applicable^†^

Domain	Variable name	Item content	Inclusion by ambulation status**	CFA standardized factor loadings^
Fatigue impact				
	fatig1	My child got tired easily	ATN	0.81
	fatig2	Being tired made it hard for my child to keep up with schoolwork	ATN	0.94
	fatig3	My child had trouble starting things because he/she was too tired	ATN	0.95
	fatig4	My child was so tired it was hard for him/her to pay attention	ATN	0.93
	fatig6	My child was too tired to enjoy the things he/she likes to do	ATN	0.89
Strength impact				
	str1	My child was strong enough to open a heavy door	ATN	0.82
	str2	My child was strong enough to pour a drink from a full pitcher or carton	ATN	0.97
	str3	My child could open a jar by himself/herself	ATN	0.88
	str4	My child was strong enough to raise his arms over his/her head	ATN	0.81
Upper extremity function				
	upper1	My child could button his/her shirt or pants	ATN	0.95
	upper2	My child could open the rings in school binders	ATN	0.95
	upper3	My child could pull a shirt on over his/her head without help	ATN	0.93
	upper4	My child could put on his/her shoes without help	ATN	0.85
	upper5	My child could use a key to unlock a door	ATN	0.94
Cognition function				
	cog1	Your child reacts slower than most people his/her age when he/she plays games	ATN	0.78
	cog2	It is hard for your child to find his/her way to a place that he/she has visited several times before	ATN	0.85
	cog3	It is hard for your child to pay attention to one thing for more than 5–10 min	ATN	0.86
	cog4	Your child has to read things several times to understand them	ATN	0.88
	cog6	It is hard for your child to understand pictures that show how to make something	ATN	0.91
	cog7	It is hard for your child to add or subtract numbers in his/her head	ATN	0.84
	cog8	Your child has to use written lists more often than other people his/her age so he/she will not forget things	ATN	0.90
	cog9	It is hard for your child to find the right words to say what he/she means	ATN	0.87
	cog10	Your child has trouble recalling the names of things	ATN	0.90
Negative affect (Bifactor model)				
Anger	affect1	My child felt mad	ATN	0.74
Anxiety	affect2	My child felt nervous	ATN	0.81
Anxiety	affect3	My child felt scared	ATN	0.79
Anxiety	affect4	My child felt worried	ATN	0.87
Anxiety	affect5	It was hard for my child to relax	ATN	0.88
Depression	affect6	My child felt lonely	ATN	0.64
Depression	affect7	My child felt sad	ATN	0.81
Depression	affect8	My child didn't care about anything	ATN	0.69
Anger	stress1	Small things upset my child	ATN	0.71
Positive affect				
	peer2	My child felt a sense of belonging around his peers	ATN	0.66
	pos1	My child felt happy	ATN	0.92
	pos3	My child was in a good mood	ATN	0.94
	pos4	My child felt calm	ATN	0.81
Mobility*				
	mob1	My child could get in and out of a car	ATN	NA
	mob2	My child could do sports and exercise that other kids his/her age could do	ATN	NA
	mob3	My child could get up from a regular toilet	ATN	NA
	mob4	My child could keep up when he/she played with other kids	ATN	NA
	mob5	My child has been physically able to do the activities he/she enjoys most	ATN	NA
	mob6	My child could get up from the floor	AT	NA
	mob7	My child could walk more than one block	AT	NA
	mob8	My child could ride a bike	AT	NA
	mob9	My child could walk across the room	AT	NA
	mob10	My child could run a mile	A	NA
	mob11	My child used a wheelchair to get around	ATN	NA
	mob12	My child used a medical scooter to get around	ATN	NA
	mob13	My child could move his/her legs	N	NA
Sleep-device symptoms*				
	sleep3	My child had muscle-cramping during the night	ATN	NA
	sleep4	My child's leg braces were uncomfortable during the night	TN	NA
	sleep5	My child was on a breathing machine at night (C-PAP or Bi-PAP)	ATN	NA
	sleep6	My child had difficulty changing positions in his sleep	TN	NA

Two different response options for the mobility items: never to almost always for all but mob11 and mob12; and with no trouble to not able to do for all others

^†^ Original PROMIS Parent Proxy items are reprinted with permission from The Assessment Center

^For the bifactor model, the loadings correspond to the general factor

*Clinimetric index

**A = Ambulatory, T = Transitional, *N* = Non-ambulatory

Two domains –mobility and sleep-device symptoms—did not fit a unidimensional model well although the standardized factor loadings were all high (i.e., ≥ 0.79, data not shown). These domains thus required substantial further modeling. Both domains were retained because caregiver-interview feedback emphasized their patient relevance.

The mobility domain’s lack of simple structure presented analytic challenges. Mplus CFA output revealed that for a number of items one could almost entirely predict the response to one item based on another. These items were, however, retained because they were needed to reflect the full spectrum of mobility disability. For example, if one requires a wheelchair to get around, one is not able to run a mile; but both extremes of the continuum need to be assessed for the content validity of the measure. This problem was not resolved by reverting to the pre-imputed mobility items (i.e., where missing values remained rather than being imputed). Iterative modeling aimed at resolving issues of residual correlations yielded a brief item set that missed content specifically noted by caregivers as relevant and important to the DMD disability experience. We tried recoding items with problematic item trace lines, collapsing five response options into two or three. This recoding did not improve model fit. We also tried modeling the three groups separately (A, T, N), but this approach also failed to yield a simple structure.

In the process, we noted that item distributions among the A group included a large subset of participants (n ~ 60) that reported their child was not able to do activities that would be expected of ambulatory individuals, such as get up from the floor or walk across the room. We thus excluded this subset from the ambulatory cohort and computed a CFA within the modified A group (n ~ 90). Further, we were able to create a unidimensional model that included six of the 13 mobility items and generated an RMSEA of 0.106. However, we considered the missing item content as well as the general multi-dimensionality and residual-correlations across A, T, and N, subgroups, and we decided that retaining all 13 items would better serve the overall goal of having a content-valid DMD-specific measure across all stages of disease severity.

Thus, for both mobility and sleep-device symptoms, we retained them as clinimetric indices [49–51] (i.e., uncalibrated scales), represented as a simple summative indices. This simple summation is justified by the abovementioned high standardized factor loadings.

### Scoring

In order to increase the accessibility of the DMD Impact Measure derived from PROMIS parent-proxy item banks, we provide two approaches for scoring the domains: (1) simple sums (i.e., raw total score); (2) IRT-based T-scores. For IRT-based scoring of all domains except sleep-device symptoms and mobility, we would recommend use of the scoring tables provided in the associated manual (available upon request). These scores use a standardized T-score metric, with a mean of 50 and a standard deviation of 10. Pearson correlation coefficients assessing the association between these two scoring approaches suggest that the simple-sum scoring yields a good estimate of the IRT-based scoring (0.95 ≤ r ≤ 0.99; Table 3). We anticipate, however, that the IRT-based scoring will be more sensitive and responsive to change.

### Application: comparison of proxy-reported domain scores by care-recipient age

**Fig. 1:**
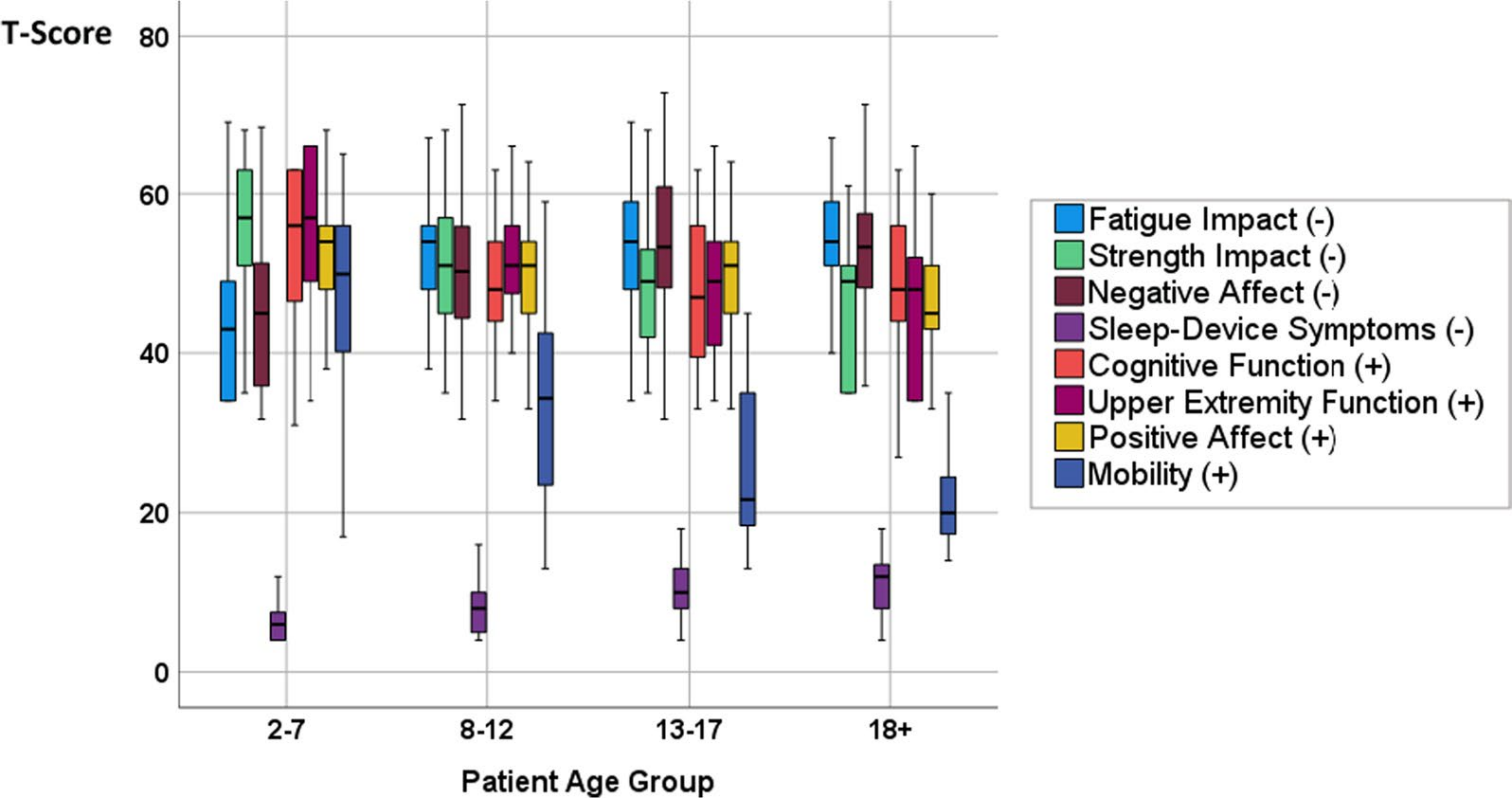
Box-and-Whiskers Plot of the Eight Parent-Proxy Domain Scores by the Four Age Groupings. This plot illustrates how the DMD-specific parent-proxy measure could be used in clinical practice. Plotting the DMD patient’s T-scores over time (y-axis) could be useful for pinpointing issues needing clinical attention. The study data show clear age-related decreases in functioning or increases in impact. Domains with largest age-related worsening were, in order of explained variance, mobility, sleep-device symptoms, fatigue impact, strength impact, upper extremity function, negative affect, positive affect, and cognitive function

As an illustration of the use of this DMD-specific parent-proxy measure, Fig. 1 shows box-and-whiskers plots for each of the eight domain T scores within each age stratum. All eight domains had age-group differences that were significant at the *p* < 0.000001 level in uni-ANOVA models, and explained variance for a given domain ranged from 0.07 to 0.47. Domains that showed the largest age-related decreases in functioning or increases in impact were, in order of explained variance, mobility, sleep-device symptoms, fatigue impact, strength impact, upper extremity function, negative affect, positive affect, and cognitive function (partial eta^2^ = 0.47, 0.28, 0.23, 0.23, 0.19, 0.08, and 0.07, respectively). Plotting the DMD patient’s scores over time could be useful for pinpointing issues needing clinical attention.

## Discussion

The present study created a DMD-specific proxy-report measure derived from PROMIS item banks that measure the full range of DMD outcomes noted as important and relevant by DMD caregivers. Validating the PROMIS Parent Proxy item sets in the DMD population was a central reason motivating this work. We do not believe that the construct validity of a disability domain would differ across patient populations, but rather that some items may be far more or far less likely to be endorsed in some patient populations. Such differences might result in dependency between items which challenges unidimensionality. Based on our analysis for example, we opted to retain more items in the mobility domain than the mobility short form, in order to clearly capture the range of mobility disability and to retain items deemed relevant by the caregivers. This decision was motivated by a desire for better content validity, while at the same time it undermined unidimensionality (i.e., high RMSEA). Mobility was thus relegated to the “clinimetric” category. Thus, construct validity for a disability domain measure is not different for those with DMD, but some items used to measure the construct may function in a different way.

The resulting measure includes eight domains that reflect the conceptualization based on caregiver input. Six of these domains had strong psychometric characteristics, and two of which are better conceptualized as clinimetric and are scored using raw sums. The resulting measure can be scored quickly and manually or using the IRT-scoring tables to provide more precise metrics. The scores resulting from the two methods are highly correlated.

The caregivers included in this study were generally parents of the person with DMD. As is the case with most childhood-onset health conditions, parents bear the greatest responsibility for their child. While siblings, other relative, and paid aides may also provide caregiving support, our study predominantly reflects parental caregivers. Future research might explicitly focus on siblings, other relatives, and paid aides in furthering the validation of this condition-specific proxy report measure of DMD disability. Additionally, the present work supports the use of the new measure in observational research, it would be worthwhile to examine the measure’s longitudinal construct validity (i.e., responsiveness [52]), as well as its usefulness in clinical work. Input from clinicians would be helpful in ascertaining how helpful this proxy-reported information is in the clinical setting.

Of note, the mobility scale posed many challenges for psychometric scale development. Most pointedly, exclusion of key items might have resolved issues of local dependence (i.e., residual correlations) but would have overlooked content specifically noted by caregivers as central to the DMD disability experience. An approach that was blinded to item content might have produced a short form that had good psychometric characteristics. However, this approach ignored the reality that most people with DMD use a wheelchair at a relatively young age and, as their disability progresses, lose the ability to walk a block, walk across the room, or get up from the floor. Dropping such items that are ‘predictable’ from an IRT perspective sacrifices content validity and possibly responsiveness to clinically important change over time.

This initial version of the DMD-specific proxy-report measure built on the Lowes Algorithm and reduced the number of items presented by about eight. The tailored administration is now only one or two items shorter than the full administration because the psychometric analyses supported removing a number of items from the final item set. Even if one were to rely only on one to two questions to determine wheelchair use to tailor the mobility and sleep-device symptoms items, the total number of items administered would be about the same in a tailored versus not-tailored administration. Thus, tailoring does not reduce the length of the measures and would not be recommended. Future research will investigate whether full administration of all items within the mobility and sleep-device symptoms domains renders any differences in terms of simple structure and model fit.

The present study had clear advantages in terms of relatively large sample sizes across the disability trajectory (~ 130 in each child age group). The data enabled careful psychometric modeling that considered relevant subgroups. The limitations of the study must be acknowledged, however. First and foremost, the study is only able to address the cross-sectional characteristics of the measure. Longitudinal construct validity [52] was not addressed, in particular responsiveness to clinically important change [53] and stability in the face of no change [54]. Second, as with any scale development, its validation is iterative. Future work should continue the validation of the new measure in an independent data set. Such future work might explicitly include a Patient and Public Involvement phase that expands the role of the DMD caregivers to be partners at all stages of the research [55–58]. Third, it would be worthwhile to investigate differential item function related to gender and education. While this investigation is beyond the scope of the present study, future work might address this important aspect of item function. Fourth, the new measure is explicitly for proxy assessment, not patient assessment. This decision was made because the study was focused on DMD caregivers, not patients. Accordingly, we did not collect patient data in conjunction with the proxy-reported measure. Future work might utilize the patient-reported versions of the proxy domains and items used, and validate this set of PROMIS scales for use in patient-reported research.

## Conclusions

In summary, we present a measure derived from the PROMIS Parent Proxy item banks for use in DMD research and clinical practice. The measure reflects content noted by DMD caregivers in qualitative interviews, and will facilitate a better understanding of how care-recipients’ disability impacts caregiver burden. Scoring metrics enable quick manual scoring and slightly more computer-intensive IRT-based scoring to fit the user’s purpose. Future research to assess the responsiveness and validity of the measure over time is warranted, to fully support its use for person-centered DMD research over the full range of patients’ ambulatory status. Future research should also address the relationship between caregiver proxy report and DMD patient self-report.

## Data Availability

The study data are confidential and thus not able to be shared.

## Abbreviations

Uni-ANOVA: Univariate Analysis of Variance
CFI: Comparative fit index
DMD: Duchenne Muscular Dystrophy
IRT: Item response theory
LLC: Limited liability corporation
PRO: Person-reported outcome
PROMIS: Patient-Reported Outcome Measurement Information System
QOL: Quality of life
RMSEA: Root mean square error of approximation
TLI: Tucker-Lewis Index

## Footnotes

1 The term “impact” is used because it is less negative than “burden”. Past research done by members of our group with caregivers of children with genetic disorders revealed that caregivers preferred the former to the latter because it allowed for positive aspects of caregiving which were broadly acknowledged by study participants [21].

## Code availability

Requests for software code will be considered, and will be made available if deemed reasonable.
